# Emerging challenges in aquaculture: Current perspectives and human health implications

**DOI:** 10.14202/vetworld.2025.15-28

**Published:** 2025-01-09

**Authors:** M. Oghenebrorhie Ruben, A. Bolanle Akinsanola, M. Ekemini Okon, Teslim Shitu, I. Iretomiwa Jagunna

**Affiliations:** 1 Landmark University SDG 2 (Zero Hunger), Landmark University, Omu-Aran, Nigeria; 2 Department of Animal Science, Landmark University, Omu-Aran, Nigeria; 3 Department of Food Science and Microbiology, Landmark University, Omu-Aran, Nigeria; 4 Department of Animal Sciences and Aquatic Ecology, Faculty of Bioscience Engineering, Ghent University, Ghent, Belgium; 5 Department of Microbiology, University of Ilorin, Kwara State, Nigeria

**Keywords:** antibiotic resistance, aquaculture effluent, eutrophication, food safety, genetic engineering

## Abstract

Aquaculture, the cultivation of aquatic organisms for human consumption, has become an essential contributor to global food security. However, it faces numerous challenges that threaten its sustainability and capacity to meet the growing demand for animal protein. This review investigates these challenges, with a particular focus on environmental degradation, public health risks, and ethical dilemmas posed by genetic interventions in fish breeding. Despite the promise of genetically modified organisms (GMOs) in enhancing fish production, their integration into aquaculture remains controversial due to potential risks and unresolved ethical questions. This study aims to provide a comprehensive understanding of these pressing issues and propose pathways for sustainable aquaculture development. With the global population increasing and the demand for animal protein intensifying, aquaculture holds great potential as a sustainable food source. However, its contribution to global protein demand remains minimal, projected to decline to as low as 4% in the coming decades. Furthermore, aquaculture’s environmental impact, including pollution of water bodies and ecosystem disruption, poses serious threats to biodiversity and public health. Addressing these challenges is critical for ensuring the long-term viability of aquaculture. By exploring the intersection of sustainability, ethics, and innovation, this review provides valuable insights for policymakers, industry stakeholders, and researchers seeking to advance sustainable aquaculture practices. This study aims to evaluate the current state of aquaculture and identify key challenges related to environmental sustainability, public health, and ethical considerations. It seeks to explore the potential of sustainable practices and genetic interventions to address these challenges while balancing the need for increased production and societal acceptance. The ultimate goal is to offer practical recommendations for fostering a resilient and ethical aquaculture industry capable of meeting future global food demands.

## INTRODUCTION

Aquaculture production has significantly increased globally in recent years. Global aquaculture growth is driven by the market for its products and by-products due to their increasing demand from the ever-increasing global human population [1, 2]. In addition to population rise, economic growth and increased income are often accompanied by a shift in dietary needs, resulting in increased demand for fish and fish products. However, concerns about food security are of great importance, especially in developing countries with a high percentage of the global population [3–6]. Thus, access to food and a sound healthcare system is necessary for every human being to survive because it affects society’s economic and social health. The lack of access to good food, particularly of aquatic sources, is an increasing concern [4, 7]. Although aquaculture is rapidly increasing the animal-source food supply globally, it has already been attracting controversy for its adverse environmental effects globally [8–11].

These issues encompass the environment, fish diseases, socioeconomics, advances in aquaculture technology, fish nutrition, perceptions about fish health and welfare, biotechnology, and the exploration of new aquaculture species [12]. In marine aquaculture, studies have reported concerns about the environmental footprint, natural resource protection, animal growth, and species diversification [13–15]. The effects of pollutants from maritime oil spills on young fish, temperature changes, and the introduction of new species are some of the specific challenges faced by marine aquaculture [16]. Furthermore, to maintain sustainable aquaculture in the Asia-Pacific area, issues such as infections, antibiotic resistance, the effects of climate change, food safety, and market accessibility must be addressed [17]. Dealing with these challenges requires a multidisciplinary approach to secure the aquaculture industry’s long-term development, given the increasing global human population. The aquaculture sector must also innovate and anticipate the challenges to reach its full potential and deliver sustainable and equitable aquatic food in the future.

In this review, our aim is to evaluate the current state of aquaculture and identify key challenges related to environmental sustainability, public health, and ethical considerations, explore the potential of sustainable practices and genetic interventions to address these challenges while balancing the need for increased production and societal acceptance. On this note, make recommendations for future perspectives as we drive for more sustainable production, supply, and adequate aquatic-source food supply globally.

## AQUACULTURE AND FUTURE GLOBAL PROTEIN NEEDS

Protein is a vital component of food ingredients eaten by humans and fish. Over the years, it has been identified as a major source of global protein. Since 1961, global consumption of aquatic foods (other than algae) has expanded at a 3.0% annual rate, exceeding population growth at a 1.6% annual rate. Consumption per person of aquatic food increased from 9.9 kg on average in the 1960s. It reached a record high of 20.5 kg in 2019, while in 2020, weight marginally decreased to 20.2 kg. Increasing earnings and urbanization, as well as enhanced post-harvest procedures and nutritional patterns, are expected to shift, driving a 15% increase in aquatic food consumed in 2030 [18].

Comparatively, the global meat supply is projected to reach 377 Mt by 2031, while fisheries and aquaculture supply is forecast to reach 203 Mt by this period [19]. According to Boyd *et al*. [13], animal protein from land animals and their products contributed 76,966 Kt of crude protein, whereas 13,950 Kt and 15.3% of animal protein from aquatic animals contributed in 2018. Capture fisheries contributed 7135 Kt of crude protein, whereas aquaculture yielded 6815 Kt. With the global protein demand standing at approximately 202 Mt in 2010, meat contributed (18%), dairy (10%), fish and shellfish (6%), and other animal products (9%), with the bulk of it coming from plant protein [20]. The average per capita protein consumption is forecast to increase by 4%, reaching 87 g/person/day in 2031, with plant protein contributing over 80% [19].

According to these statistics, the contribution from animal protein in the period under study (2022–2031) will be <20%, with the contribution from fish forecasted to be about 4%. Income-related issues, cultural issues, health and environmental concerns, and ethical considerations regarding animal welfare and consumption are the main drivers. However, regular meat consumption has been documented to increase the risk of certain non-communicable diseases (NCDs), such as heart disease, pneumonia, bowel cancer, and etcetera [21].

Furthermore, it has been established that diets derived from fish products and their by-products are some of the health-friendly diets with less environmental impact [22, 23]. To sustainably increase fish food production to meet rising demand, there is a need to develop new strategies for food security at national, regional, and global levels, thereby enhancing the contribution of fish and fisheries products to the continuous transformation of food systems and ensuring the abolition of hunger and malnutrition.

## AQUACULTURE AND ENVIRONMENTAL ISSUES

Water pollution has greatly threatened aquatic organisms, plants, humans, and the climate [24]. Although aquaculture increases the amount of animal protein required to meet global demand, it has not been without negative blowbacks. Over the years, there have been increasing concerns about the effects of fish farming on the environment [13–15]. Wastewater from aquaculture is most often released into the environment without treatment and finds its way into the natural surface, groundwater bodies, and surrounding soils [25].

In aquaculture, especially fish farming, wastewater is burdened with nutrients from fish feces and uneaten feed, and chemicals are the effluents that end up in the environment. Chemicals such as therapeutics, disinfectants, anesthetics, and compounds used in water/fish treatment, especially in areas with poor water quality, when not treated before disposal, cause serious pollution of natural water bodies and soil, an example of which is the eutrophication of surface water bodies [26, 27].

## EUTROPHICATION

Eutrophication is the most significant cause of depletion of surface water quality and, in most cases, results in hypoxia [28, 29]. During photosynthesis, aquatic plants (algae) use carbon dioxide to produce oxygen. However, when these algae complete their life cycle, they die, sink, and eventually decompose. During this process, bacteria feed on them and use up the available oxygen in the water, thereby decreasing the oxygen for other aquatic life in the water body. In addition, when these algae sink to the bottom of the water, the oxygen in sulfates (SO_4_^2-^) is also used up by specific bacteria, resulting in sulfur release (S^2-^) [30, 31]. This immediately captures the oxygen that is still available in the upper stratum. In addition, algae blooms screen the entire water surface, making it impossible for atmospheric oxygen to be absorbed into the water body. In addition, an increase in algae biomass leads to an increased rate of photosynthesis and corresponding depletion of dissolved inorganic carbon, thereby raising the pH to extreme levels during the day [30], which may result in the death of aquatic life. In addition, increased pH levels have been documented in blind organisms that rely on the perception of dissolved chemical cues for survival [32].

The increase in algae, particularly macroalgae, phytoplankton (diatoms, dinoflagellates, chlorophytes), and cyanobacteria, can discharge poisonous substances into the water or be poisonous themselves. These toxins, for example, from cyanobacteria, have been documented to primarily affect the nervous system and liver [33] and are poisonous to humans (mainly through drinking water) [34]. Another effect of eutrophication is the change in zooplankton, shellfish, and finfish populations due to the depletion of oxygen and increased pH level of the water body, resulting in massive die-offs of these organisms [28, 29].

## AQUACULTURE WASTEWATER

Aquaculture wastewater has been documented to contaminate groundwater, altering water chemistry and making it unfit for drinking [35]. Gallegos *et al*. [36] reported increased nitrate levels in groundwater in all Mezquital Valley groundwater due to wastewater irrigation, although this was not specific to aquaculture wastewater. According to Elemile *et al*. [37], anthropogenic activities have a major influence on the quality of groundwater. In addition, the bulk load of microbes that contaminate groundwater comes from animal and human fecal and effluent from fish farms are not excluded from the study.

In the same vein, aquaculture wastewater has a negative impact on soil life, particularly in arid regions. Studies have found that aquaculture farm wastewater for irrigating soil can reduce microbial communities, which play important roles in soil health. This is mainly due to the increased salinity of soil, especially from chloride (Cl) levels [38, 39]. Fish farmers adopt various medication regimes for different reasons, from therapeutic to prophylactic and growth-promoting. Boxall [40] reported low-to-high concentrations of antimicrobials, hormones, disinfectants, and steroids in soils and waters (surface and ground). These chemicals have been reported to destroy soil biotopes, microbial function, and soil biodiversity [39, 41]. Furthermore, these chemicals and the application of limes in aquaculture ponds have been reported to alter the physical and chemical characteristics of soil, which could also increase the problem [42].

Similarly, wastewater from shrimp culture facilities is high in nutrients, heavy metals, and other noxious substances [43]. When such effluents are used constantly for irrigation, they affect crops and consequently the consumers of such crops [44].

Other negative impacts include salinization and acidification of the surrounding soil [45]. Ahmed *et al*. [46] reported 50%–90% carbon storage in soils receiving wastewater from shrimp farms and attributed this to increased salinity.

## DESTRUCTION OF IMPORTANT ECOSYSTEMS AND HABITATS

In addition, another harmful impact of aquaculture on the environment is the destruction of important ecosystems and habitats [42, 47]. This is often the case when key ecological areas, such as mangrove forests, are converted for aquaculture. Mangrove forests are keystone resources and home to corals, seagrass beds, and nursery grounds for many fish species. They strengthen the coastline, and the trees serve as pollutant and sediment traps [48, 49].

**Figure 1:**
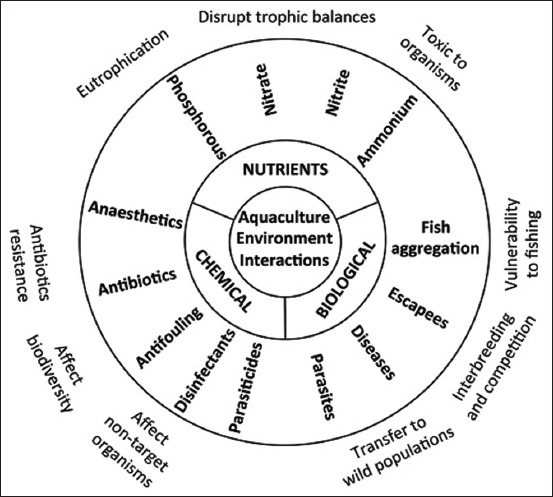
The main pollution sources of marine finfish aquaculture and their related effects on the environment [26].

The deforestation of mangrove forests for the farming of fish, especially shrimps, has been documented as a cause of significant habitat loss [50, 51]. In their study [51], the authors documented that 35% of the global mangrove forest area had been lost due to anthropogenic activities such as fish farming and salt pond development. In addition, several countries have recently reported a decline in global mangrove cover. Hagger *et al*. [52] reported that mangrove cover declined by 35% by the end of the 1990s and by a further 2.1% between 2000 and 2016. This study attributed the decline in mangrove conversion to aquaculture and agriculture. Figure 1 [26] shows the main sources of pollution from marine aquaculture.

## AQUACULTURE-FISHERIES INTERACTION

Aquaculture has a strong correlation with fisheries, as it impacts aquaculture both positively and negatively. Aquaculture has been reported to impact fisheries through the entire production process as inputs (seed and feed), resource use (land and water), and outputs (excess nutrients and escapee). We have discussed some of these routes in aquaculture and the environment. In this section, how the use of fisheries resources as input (seed and feed) and its impact on fisheries are discussed.

## CAPTURE-BASED AQUACULTURE (CBA)

The culture of some fish species is dependent on the capture of seeds from the wild and then stocking and rearing for human use. This system is primarily adopted for species that are difficult to breed in captivity. In Soto *et al*. [53], an expert review on addressing aquaculture-fisheries interactions through the implementation of the ecosystem approach to aquaculture reported that about 70 species of fish are included in the CBA. The examples are the majority of farmed mussels, some farmed shrimp (*Penaeus monodon* and, some freshwater prawns). Basic finfish in this category include tuna, mullet, cod, carp, groupers, and others [54]. Table 1 shows the major fish groups used in the CBA and their origins.

**Table 1:** Main species groups in the CBA and global estimated proportions of seed origin.

Species group	Source of larvae
Bluefin tuna	Wild
Eels	Wild
Oysters	Mostly wild (<10% from hatchery)
Mussels	Mostly wild (<10% from hatchery)
Lobster	Mostly wild (<10% from hatchery)
Seahorse	Mostly wild (<10% from hatchery)
Mullet	Mostly wild (<10% from hatchery)
Cod	Half of the hatchery
Grouper	Half of the hatchery
Sea cucumber	Half of the hatchery
Shrimp	Mostly hatchery
Tilapia	Mostly hatchery^2^
Carps	Mostly hatchery

Source: FAO internal aquaculture database, CBA=Capture-based aquaculture

Some of the negative effects of aquaculture dependence on fisheries for seed use are presented in Table 2.

**Table 2:** Negative impacts of CBA [55].

S. No.	Impacts and signs of effects
1	Mass capture of wild seeds, juveniles, and broodstock can lead to the negative recruitment of wild fisheries
2	Bycatch of other species and target species can lead to biodiversity loss, potentially affecting wild fisheries.
3	Destructive fishing practices for the collection of wild seeds or broodstock (-) can damage fisheries’ habitats.

CBA=Capture-based aquaculture

## ISSUES STEMMING FROM AQUACULTURE’S DEPENDENCE ON FISHERIES FOR FISHMEAL AND FISH OIL

Furthermore, with the high demand for seafood and aquatic resources, the aquaculture industry is intensified and the demand for fishmeal and fish oil. This has resulted in a consistent reliance on wild resources for fishmeal and fish oil for fish feed, resulting in the overburdening of captured fisheries. According to the State of the World Aquaculture and Fisheries report for 2020 [18], over 20 million tons of total world fisheries and aquaculture catch was used for purposes other than food, of which 16 million tons was used in the production of fishmeal and fish oil. In addition, by-products account for more than 27% of global fishmeal output and 48% of overall fish oil production.

Concerns have been expressed regarding the influence of aquaculture on capture catches due to potential consequences on wild fish populations and the environment. Aquaculture has expanded to meet the growing demand for seafood as worldwide demand has surged and capture fisheries have stalled. However, this increase has been linked to negative environmental consequences, such as pollutant discharge from fish farms into coastal seas [56]. Furthermore, the expansion of aquaculture is projected to affect the availability and demand for wild fish, thereby affecting the welfare of fishing consumers and producers [57]. Aquaculture’s overall effects on capture catches and the environment are still unclear, even if it might help offset the decline in the availability of wild fish [57]. Sustainable techniques are thus needed to reduce aquaculture’s harmful effects on wild fish populations and the environment. Table 3 [53] presents some negative issues arising from the reliance on fisheries for fishmeal and fish oil.

**Table 3:** Issues related to the use of fish as aquaculture feed [53].

S. No.	Impacts and signs of effects
1	Increased pressure on pelagic fisheries resources that provide fishmeal and fish oil
2	Increased fishing pressure on low-value fish that feed directly to higher-value fish
3	Low value” fish taken out of the market chain for hungry and poor
4	The price of low-value fish rises due to demand from aquaculture, making them less accessible to the poor and hungry.

## AQUACULTURE AND PUBLIC HEALTH ISSUES

Aquaculture is becoming the world’s leading food-producing sector, with rapid growth occurring [58, 59]. Seafood is a significant source of good dietary fats [60]. Aquaculture produces almost half of the seafood consumed worldwide [61]. However, to increase productivity, several chemicals (including probiotics, antibiotics, disinfectants, herbicides, and pesticides) are used in aquaculture to cure and prevent diseases [62]. Due to the use of these chemicals, contaminated farmed seafood items pose dangers to human health, possibly increasing the number of various NCDs like cancer [63–66]. In aquaculture, antibiotics are used as preventative, therapeutic, or feed supplements. Antibiotics can disperse into the water column through treated feed and spread to sediments and wildlife [61].

In comparison with terrestrial animal farms, aquaculture requires intense culture methods and antibiotics. As a result, nearly 80% of the antibiotics and metals that are applied in the aquaculture industry end up uneaten in medicated feeds; unabsorbed antibiotics and waste discharged from the cultured life form find their way into the soil and aquatic environments near aquaculture facilities. As a result, a favorable environment for establishing and enriching persistent aquatic antibiotic-resistant genes (ARGs) has been created [67, 68].

Antibiotics are typically administered to aquaculture animals through surface-coated or pelleted diets and other water immersion or injection methods. Today, the use of antimicrobials by both humans and non-human animals has led to the emergence and spread of antimicrobial resistance as a global public health issue. In addition, the use of antibiotics in one ecological niche, like aquaculture, can affect the level of resistance in another ecological niche, such as human medicine, and resistance issues in one country can be transferred to another one [58, 69, 70].

Worldwide use of antibiotics in food animals, including fish farming, is expected to increase 67% by 2030, from an estimated 63,151 tons in 2010 [71]. Aquaculture does not always use antibiotics responsibly, and restrictions on their usage have not adequately guaranteed the avoidance of dangers to humans. Veterinary oversight of farmers’ antibiotic administration while adhering to withdrawal periods before slaughter, correct dealers’ distribution and handling, and clear instructions from drug makers are necessary for responsible antibiotic use.

Concerns about food safety and the health of consumers, which are generally neglected in most underdeveloped countries, could arise from the unregulated application of antibiotics in the aquaculture industry to produce shrimp and fish on farms [71]. Even at extremely low concentrations, antibiotic residues can be found in the treated animal’s edible tissues due to their use in food-producing animals. The presence of antibiotic residue in fish muscles may be the result of incorrectly following label instructions or dosage recommendations, skipping documented withdrawal periods, overdosing at a single injection site, using equipment tainted with antibiotics, failing to clean equipment used for drug preparation or delivery; mixing mistakes; inadvertent feeding of chemicals spilled on surfaces; or medicated diets. Additional causes include drug interactions, changes in water temperature for aquatic animals, environmental contamination, age, pregnancy, congenital conditions, diseases, and allergies in animals, and incorrect drug use [72].

The widespread use of antibiotics in aquaculture has become a pressing issue posing a threat to health, environmental sustainability, and the integrity of the food system. Antibiotic residues found in aquaculture products directly endanger health because they accumulate in the food chain and cause adverse health effects. For instance, chloramphenicol residues are associated with an increased risk of cancer, and even low concentrations can cause anemia. This life-threatening condition disrupts the production of leukocytes. Furthermore, other antibiotic residues can lead to carcinogenicity, mutagenicity, nephropathy, and allergies [73].

The uncontrolled use of antibiotics in aquaculture also disturbs the balance within ecosystems by promoting the growth of antibiotic-resistant bacteria and potentially harmful changes to the microbial communities that support marine life. This ecological disturbance can have far-reaching consequences for health because antibiotic-resistant bacteria pose significant challenges in treating human infections. The impact of residues in aquaculture extends beyond well-being by affecting the collective health, environmental stability, and economic viability of aquaculture industries. As a result, it is crucial to tackle this problem through a strategy that includes more stringent regulations, enhanced aquaculture methods, and public awareness initiatives [58]. FAO/WHO/OIE identified the gaps and needs for future research to reduce the use of antibiotics in aquaculture, including the need for more information on seafood consumption in various regions and among different subpopulations and the spread of resistance genes from fish and aquatic bacteria to human pathogens [58].

The impact of aquaculture on the environment can be negative for coastal towns by altering citizens’ sense of place, reducing civic engagement, and deteriorating mental health. In addition, the use of fish in aquaculture decreases the quantity of fish available for human consumption [61]. Using fish as a core feed ingredient for fish production causes the food-feed trade-off in coastal and island environments, particularly in low-income countries where fish and other seafood provide a major portion of the population’s necessary animal protein and small fish serve as their principal source of micronutrients [74]. Hence, aquaculture may negatively influence human health and nutrition by depleting wild fish populations, adversely affecting the environment, and spreading fish diseases that will lower the production of fisheries or aquaculture in the future. Food security could be affected by a lack of wild fish and/or aquaculture products, which might lead to an increase in the consumption of foods that encourage the development of NCDs [61].

Furthermore, wastewater containing antibiotic residues and ARGs is frequently treated to produce aquaculture sludge, which is used as an organic fertilizer, or flows into aquatic habitats following treatment. Treating aquaculture effluents and applying aquaculture sludge as organic fertilizer to the soil can generate a conduit for ARGs to travel from animals to soils and crops. This may impact urban dwellers, agricultural customers, and downstream occupational workers [75, 76]. However, note that most developing nations lack regulations and/or insufficient waste treatment systems for aquaculture. The risk of transmitting ARGs from aquatic ecosystems to humans may increase if untreated aquaculture effluent directly enters adjacent water bodies [77].

## AQUACULTURE AND FOOD SAFETY ISSUES

Worldwide, marked morbidity and mortality have been documented to be caused by foodborne diseases (FBDs) due to the ingestion of hazardous contaminated food [78–80]. Hazards in food include biological hazards (bacteria, parasites, fungi, and viruses) and chemical hazards (agrochemicals, metals, antibiotics, metals, organic pollutants, and feed additives). According to Lake *et al*. [81], 1 in 10 persons annually, accounting for over 600 million people globally, suffers from FBD. The World Health Organization estimates that 33 million years of healthy lives are lost each year due to the consumption of unsafe food. Thirty percentages of these deaths were recorded in children under the age of 5 [82]. As a food-producing sector, aquaculture systems are rapidly growing as the demand for aquaculture products increases. An estimated 3.3 billion people in the world consume at least 20% of their animal protein intake from finfish and shellfish [18].

**Figure 2:**
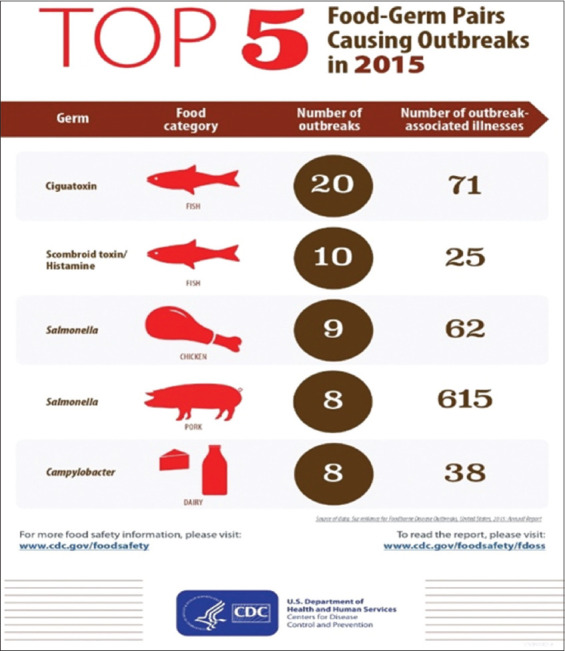
Top 5 food-germ pairs (Outbreaks vs. Illnesses [88]).

The increasing human population, rising demand for animal protein, and consideration of fish as an inexpensive and rich source of animal protein and safe food have led to intensification of the aquaculture sector, resulting in the use of chemicals, steroids, antibiotics, etc., for raising fish [83]. Microorganisms are the primary source of infectious illness outbreaks in fish, other livestock, and humans. They are frequently found on the skin and gills, liver, spleen, digestive system, and kidney. Furthermore, ample evidence exists regarding the potential foodborne risks linked to the aquaculture sector [76, 84, 85]. These risks are attributed to the ingestion of fish and their products that are not well cooked or raw, as well as the toxins produced by microorganisms and vasoactive histamines [86–89]. According to Dewey-Mattia *et al*. [90], the five top food germ pairs that caused disease outbreaks in the US in 2015 and the number of outbreak-associated diseases are shown in Figure 2 [88]. Thirty outbreaks associated with 96 illnesses were of the food category fish.

**Figure 3:**
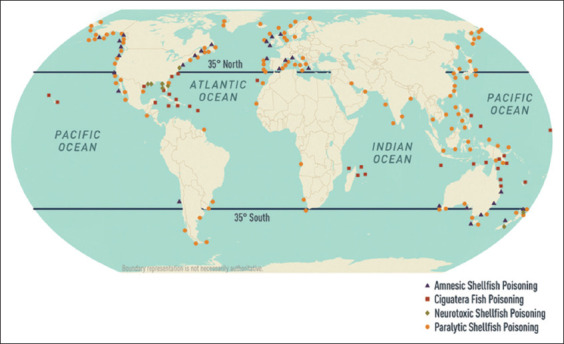
World distribution of selected seafood poisoning [93].

Furthermore, previous studies have shown that some fish and shellfish contain biotoxins that are not destroyed by heat at certain times of the year. Ciguatera fish poisoning can be caused by fish such as groupers, barracudas, moray eels, sturgeons, sea bass, red snapper, amberjack, mackerel, parrot, surgeonfish, and triggerfish. In humans, ciguatera fish poisoning is linked to various disease conditions (gastrointestinal, neurological, and cardiovascular abnormalities) and exhibits different signs and symptoms. Tetrodotoxin, sometimes known as pufferfish or fugu poisoning, and scombroid toxicity are two more prevalent poisons found in fish [91–94]. Figure 3 [93] presents the global distribution of selected seafood toxins according to the Centers for Disease Control and Prevention and Nemhauser [95].

In addition, feed fed to fish in intensive fish systems, known as complete diets (commercial feed and even locally compounded diets), is a source of contaminants such as microbes, steroids, and antibiotics [96]. These contaminants, especially microbes and antibiotics, not only cause harm to the cultured fish but also pass down from the value chain to the consumers of the fish.

Aquaculture-rearing systems have evolved over time to include wastewater-fed ponds, paddy rice-cum-fish farms, and more intensive fish-farming systems [97]. All these rearing systems have a negative impact, and products obtained from them can cause consumer FBDs. Although fish farming in wastewater-fed fish farm systems is inexpensive to produce and attain market size in a timely manner, this poses a severe threat to consumers.

Bacteria present in fecal samples can be easily transferred to the fish and then to the consumer. In addition, when raising fish using an integrated rice-fish farming system, the fish can quickly ingest the chemicals used in rice production. Problems associated with the intensive fish farming system have been well discussed in the previous paragraph, including dependence on complete feed, antibiotics, and other chemicals that threaten consumers.

**Figure 4:**
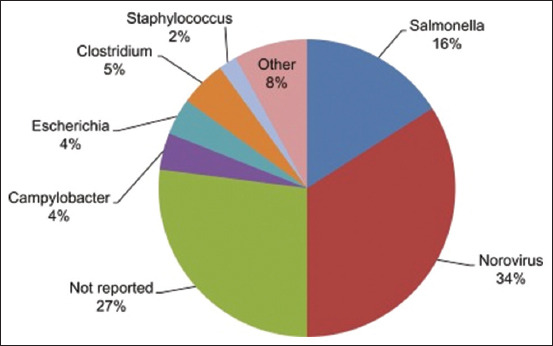
Etiology of foodborne disease outbreaks in the United States from 2008 to 2017 [101].

Foodborne outbreaks have been documented worldwide because people eat tainted foods [98, 99]. Nevertheless, prior outbreaks were associated with chemical pollutants, including mercury poisoning in Iraq, polychlorinated biphenyl poisoning in Taiwan, and the Minamata, Itai-itai, and Yusho diseases. Foodborne outbreaks in recent times have been linked to bacteria such as the *Salmonella* Typhimurium DT 104 global outbreak, *Escherichia coli* infection in Germany, paragonimiasis in Northeast India, hepatitis-A outbreak in China, and listeriosis in South Africa) [99, 100]. Jones [101] reported that Norovirus, *Salmonella* spp. *Staphylococcus*, and most microorganisms that caused FBD outbreaks in the United States from 2008 to 2017 are unreported (Figure 4) [101]. It is important, however, to report cases and the etiologies of FBDs to prevent future outbreaks.

## GENETIC MODIFICATION (GM) IN AQUACULTURE AND ETHICAL ISSUES

The intensification of the aquaculture industry due to the growing demand for fish for human and animal consumption has led to the use of various technologies to curb challenges such as parasitic and infectious diseases, limited viability, decreased fertility, and delayed growth in a bid to increase the performance of cultured fish. One such technology is Genetic Engineering (GE). GE strategies that have been applied in aquaculture to curb some of these problems include DNA vaccines, transgenes in fish, GM feed, and GM plants as edible vaccines [102, 103]. GM AquAdvantage salmon (AAS) was introduced for use in Canada in 2016. The United States in 2019 [104], as well as the Nile tilapia (*Oreochromis niloticus*) in Argentina in 2018 [104, 105], proved that GE can solve some of these problems. To generate AAS, the Chinook salmon (*Oncorhynchus tshawytscha*) growth hormone gene was placed under the promoter of an Ocean pout (*Macrozoarces americanus*) anti-freeze protein, which was subsequently inserted into an Atlantic salmon (*Salmo salar*) egg [106]. Clustered, regularly interspaced short palindromic repeats/CRISPR-associated protein 9 (CRISPR/Cas9) has been used as a current genome editing (GE) technology to modify the tilapia genome [105]. Table 4 [107] presents some experimental and developmental work on transgenic technology (genetically modified organisms [GMOs]) in aquatic species.

**Table 4:** Experimental and developmental work on transgenic technology (GMOs) in aquatic species [107].

Desired trait	Species	Active genes
Better growth (faster, bigger, more efficient)	Salmon species: chinook salmon (*Oncorhynchus tshawytscha*), Atlantic salmon (*Salmo salar*), coho salmon (*Oncorhynchus kisutch*), rainbow trout, cutthroat trout, Oreochromis niloticus, hybrids of tilapia, mud loach, channel catfish, common carp, Indian major carps, goldfish, abalone, Pacific oyster,	Growth hormone, antifreeze protein gene, insulin-like growth factor
Increased cold tolerance	Atlantic salmon, strawberries, and potatoes	Antifreeze protein gene
Increased tolerance to low oxygen levels	Common carp and grass carp	Antifreeze protein gene
Disease resistance	Salmon spp: chinook salmon (*Oncorhynchus tshawytscha*), Atlantic salmon (*Salmo salar*), coho salmon (*Oncorhynchus kisutch*) , striped bass, and marine shrimp	The lysosome and pleurocidin (flounder) genes
Sterility	Oysters, medaka	Interference RNA
Pigment synthesis	Marine bacteria	Beta carotene gene
Production of human insulin	Tilapia	Insulin-producing gene
Calcitronin production	Rabbit	Salmon calcitonin-producing genes

GMO=Genetically modified organisms

The most commonly targeted features of GE in fish farming, according to Blix *et al*. [105], Chen *et al*. [108], Datsomor *et al*. [109], Gratacap *et al*. [110], and Straume *et al*. [111], are growth, disease resistance, pigmentation, reproduction, and development, use of trans-GFP in research, and omega-3 metabolism. The most popular GE technology is CRISPR/Cas due to its simplicity, inexpensiveness, and effectiveness [112]. In addition to the changes in tilapia caused by CRISPR/Cas9, CRISPR/Cas9 is employed to alter other features in various species. The three most frequently altered species are medaka, zebrafish (*Danio rerio*), Atlantic salmon, [113], and Nile tilapi*a (Oreochromis niloticus)* [105]. The modification of genes that resist disease in grass carp (*Ctenopharyngodon idella* [114], channel catfish [115], and farmed carp [116] are other applications of CRISPR/Cas in various economically significant fish species.

Irrespective of the benefits associated with this technology, several concerns have been raised about the ecological and genetic consequences of the purposeful or unintentional release of GMOs into the environment [117–119]. GMOs may pose the following risks: the creation of new or more vigorous pests and pathogens; exacerbation of the effects of existing pests through hybridization with related transgenic organisms; and harm to non-target species, which could include soil organisms, non-pest insects, birds, and other animals. Disruption of biotic communities, including agroecosystems, and irreversible loss or changes in species or genetic diversity within species, are other likely risks.

GM feed, when broadcast in aquatic environments, can be fed to other aquatic animals, resulting in horizontal gene transfer. This may also occur from the DNA in vaccines to the recipient genome. According to Ghimire *et al*. [102], gene instability, biodiversity loss, and environmental effects on non-target animals such as fish, worms, bees, and insects have been attributed to GM plants and their environmental consequences. One of the main issues with using GMOs is that once they are accidentally or purposely released into the environment, it can be difficult or impossible to control them. As a result, any harmful products produced by these organisms continue to grow and multiply throughout their lifespan and remain metabolically active [120, 121]. Transgenic fish stocks that have been released are believed to pose a risk not only to their members of the same species but also to other species through niche extension and even speciation. The general consensus is that accidental releases of farmed fish into natural environments should be avoided, especially if the fish are GM [122]. The issue of live competition between transgenic stocks and wild populations in the environment is of particular concern because transgenic species increase fish population predation.

Naturally, the risk of a transgenic or GMO escaping is substantially higher. Due to this, most nations have stringent laws requiring rigorous risk assessments before releasing these creatures into the wild or their introduction into the market [122]. The Net Fitness Approach, which estimates essential fitness metrics for transgenic fish compared with wild-type fish and incorporates them into a model to forecast risk, can address risk assessment. The net fitness parameters are age at sexual maturity, viability to sexual maturity, mating success, fecundity, fertility, and lifespan. This makes it easier for regulators to create a set of clear risk-evaluation tests.

## CONCLUSION

The aquaculture industry plays a vital role in addressing global food security and meeting the rising demand for fish and animal protein, yet its current practices face substantial challenges. Unsustainable methods dominate the sector, leading to significant environmental degradation, including nutrient pollution, reduced oxygen levels in water bodies, and soil contamination from untreated effluents. These practices not only threaten ecosystems but also impact groundwater and soil biodiversity, further exacerbating environmental instability. Additionally, the intensification of aquaculture has led to the excessive use of harmful chemicals, steroids, and antibiotics, contributing to public health risks such as antimicrobial resistance and increased human morbidity. While genetic engineering offers potential solutions to enhance the quality and quantity of aquatic resources, it introduces ethical and ecological concerns that demand careful consideration. To sustain aquaculture’s contribution to global protein needs, it is imperative to adopt environmentally friendly practices, regulate harmful chemical use, and ensure innovations align with ecological and public health priorities.

## AUTHORS’ CONTRIBUTIONS

MOR and IIJ: Conceptualized and designed the study. IIJ, BA, and TS: Wrote the first draft. MOR and MEO: Reviewed and revised the manuscript. All authors have read and approved the final manuscript.
